# Transcriptomic Survey of How Acetate Addition Affected the Growth in *Nannochloropsis oceanica* (Suda & Miyashita) R. E. Lee

**DOI:** 10.3390/life15091398

**Published:** 2025-09-03

**Authors:** Yikai Wu, Han Zhu, Hang Su, Li Wei

**Affiliations:** 1 Ministry of Education Key Laboratory for Ecology of Tropical Islands, Key Laboratory of Tropical Animal and Plant Ecology of Hainan Province, College of Life Sciences, Hainan Normal University, Haikou 571158, China; wyk20120814@163.com (Y.W.); zh15955035863@outlook.com (H.Z.); 920515@hainnu.edu.cn (H.S.); 2 International Science and Technology Cooperation Laboratory for Marine Microalgae Ecological Carbon Sinks, Hainan Normal University, Haikou 571158, China; 3 Hainan Observation and Research Station of Dongzhaigang Mangrove Wetland Ecosystem, Haikou 571129, China

**Keywords:** sodium acetate, marine microalga, adaptation mechanism, transcriptomic, metabolic reprogramming

## Abstract

*Nannochloropsis oceanica* (Suda & Miyashita) R. E. Lee holds considerable potential for the production of high-value compounds, including pigments, lipids, and polyunsaturated fatty acids. Sodium acetate, a widely used carbon source in microbial cultivation, is both cost-effective and efficient. Although it has been reported to enhance biomass production in various microalgae, its effects on metabolic pathways differ substantially across species. In this study, we investigated the transcriptional responses of *N. oceanica* to sodium acetate supplementation using high-throughput mRNA sequencing. Sodium acetate significantly promoted growth but elicited a distinct metabolic reprogramming in contrast to patterns commonly observed in other microalgae. We identified 747 differentially expressed genes (399 upregulated and 348 downregulated), reflecting a substantial transcriptomic shift. Pathways related to lipid metabolism, carbon fixation, and photosynthesis were markedly suppressed. Notably, genes associated with photosynthesis were downregulated by 34–43 fold, suggesting a strategic reallocation of resources away from energy-intensive photosynthetic processes in the presence of an external organic carbon source. In sharp contrast to *Chlamydomonas reinhardtii* P. A. Dangear and *Haematococcus pluvialis* (Flotow) Wille, lipid metabolism in *N. oceanica* was not enhanced under sodium acetate supplementation. Instead, expression of lipid metabolism genes decreased by 5–14 fold, with most fatty acid- and lipase-related genes also downregulated (4–30 fold). Together, these findings reveal that *N. oceanica* adopts a unique adaptive strategy, channeling acetate-derived carbon primarily into rapid biomass accumulation rather than energy storage or high-value metabolite synthesis. This work provides new insights into the species-specific responses of microalgae to organic carbon sources.

## 1. Introduction

Microalgae, as a valuable biological resource, have garnered significant attention from researchers. Studies have shown that the incorporation of various organic carbon sources can influence the composition of lipids and starch in microalgae [1]. For example, in *Crypthecodinium*, glucose has been found to promote starch accumulation, while sodium acetate inhibits glucose utilization and starch accumulation, leading to a notable increase in total fatty acid production [2]. Additionally, *Chlorella zofingiensis* efficiently utilizes monosaccharides such as glucose, fructose, and mannose under dark conditions [3]. Sucrose has also been absorbed and utilized by *Chlorella zofingiensis*, promoting both algal cell growth and lipid accumulation. This microalga is capable of using lactose and starch as sole carbon and energy sources and can grow and produce astaxanthin in the dark, though its specific growth rates and cell dry masses are slightly lower compared to treatments with glucose or mannose [4]. These findings suggest that different carbon sources drive photosynthetic organisms to adjust their biosynthesis, physiological metabolism, and other processes in response to varying growth conditions.

Sodium acetate is a commonly used carbon source for many microbial species, known for its economic viability and efficiency. It promotes heterotrophic growth in *Crypthecodinium cohnii*, significantly increasing cellular biomass when compared to photoautotrophic growth conditions [5]. Similarly, in *Chlamydomonas reinhardtii* [6] and the green microalga *Haematococcus pluvialis* [7], the addition of sodium acetate increases fatty acid content in the cells. Notably, a 6-fold increase in lipid content was observed in *C. reinhardtii* when 50 mM sodium acetate was added [8]. Additionally, *Monoraphidium griffithii* showed increased cell density with sodium acetate supplementation, reaching the highest density at 0.5% [9]. Sodium acetate also enhanced total fatty acid (TFA) content in *C. reinhardtii* under phosphorus and nitrogen-deficient conditions [10]. Under nitrogen-deficient conditions, the rapid transition of *Haematococcus lacustris* into the induction phase following the addition of 1.3 or 2.6 g/L sodium acetate promoted astaxanthin accumulation [11]. Interestingly, in *Crypthecodinium* sp. SUN, the accumulation of total fatty acids is not primarily linked to the expression of key fatty acid biosynthesis genes; instead, the increase in intracellular acetyl-CoA content appears to be the critical regulatory factor [2]. In conclusion, sodium acetate plays a significant role in promoting lipid and biomass accumulation across various microalgae species.

*Nannochloropsis oceanica*, a widely distributed marine microalga, belongs to the Eustigmatophyceae classes. It can be cultured on a large scale using wastewater or grown through mixed culture methods [12]. The cells of *N. oceanica* are rich in high-value products, such as the vitamin B complex, pigments, lipids, polyunsaturated fatty acids, and terpenoid compounds, making it a valuable resource with vast potential for development [13,14]. In addition to its ability to synthesize neutral lipids for biodiesel production, *N. oceanica* can produce eicosapentaenoic acid (EPA) for functional foods. Its applications extend across various industries, including industrial, agricultural, pharmaceutical, and food sectors [15]. Furthermore, *N. oceanica*’s ability to be cultivated on a large scale outdoors, coupled with its efficient oil production and carbon fixation capabilities, has established it as one of the model industrial strains for algae [16]. Notably, the lipid production efficiency of *Nannochloropsis* is highly influenced by the carbon source used, with sodium acetate being particularly effective in enhancing lipid yield [17]. For instance, in the mixotrophic cultivation of *Nannochloropsis oculata* with 32 mM sodium acetate, the biomass and lipid yield were 1.5 times and 9.4 times higher, respectively, compared to autotrophic conditions [18]. However, the molecular mechanisms through which sodium acetate affects oil production and carbon fixation in *N. oceanica* remain poorly understood. Based on this knowledge gap, we hypothesized that sodium acetate supplementation would induce significant transcriptional reprogramming in key metabolic pathways, particularly those related to lipid synthesis, carbon metabolism, and photosynthesis, and that the response of *N. oceanica* may differ fundamentally from that of other well-studied microalgae such as *C. reinhardtii* and *H. pluvialis*. To test this hypothesis, we performed mRNA-seq to explore the regulatory effects of sodium acetate on the growth of *N. oceanica*.

In this study, mRNA-seq was employed to elucidate the regulatory effects of sodium acetate on the growth of *N. oceanica*. Differential expression analysis revealed that sodium acetate at an appropriate concentration significantly enhanced algal growth and induced extensive metabolic reprogramming along with substantial transcriptional changes. A number of differentially expressed genes were identified, particularly those involved in metabolic and photosynthetic pathways. Notably, genes associated with photosynthesis and carbon fixation were downregulated. Conversely, lipid metabolism-related genes also exhibited marked downregulation, contrasting with initial expectations. Furthermore, carbon metabolism genes showed divergent expression patterns, with both up- and down-regulation observed. These findings offer mechanistic insights into how sodium acetate influences the physiology of *N. oceanica*, contributing to a deeper understanding of carbon metabolism in marine microalgae and supporting future applications in algal genetic engineering and bioenergy research.

## 2. Materials and Methods

### 2.1. Culture Conditions of N. oceanica

For cultivation, *N. oceanica* f/2 medium was used [19]. In the experiment, to ensure reliable results, sodium acetate at a concentration of 1.0 g/L was added to the treatment group according to a specific protocol. In order to obtain meaningful data, the samples were incubated for up to 12 days to reach the plateau of the growth curve.

In the specific operation, sodium acetate was first added to the samples, and then the incubation was continued following the process of the above experimental design. Such treatment allows us to more accurately study the effects of sodium acetate on algal growth and gene expression, thus revealing the relevant life science mechanisms. Due to the long incubation period, the algae in the samples will be fully grown and amplified, and the gene expression information contained in the samples will more fully and accurately reflect the response of the algae to sodium acetate under the stable state of the plateau phase.

### 2.2. Experimental Design and Sampling

To investigate the transcriptional response of the algae to sodium acetate, we established three biological replicate treatment groups, designated AC1, AC2, and AC3, which were cultured in the presence of sodium acetate. Concurrently, three replicate control groups (C1, C2, and C3) were cultured under identical conditions without sodium acetate supplementation. For each sample, algal cells were harvested by centrifugation, and the supernatant culture medium was removed. The resulting cell pellets were temporarily stored at 4 °C prior to subsequent processing. Next, the cells were snap-frozen with liquid nitrogen and stored at −80 °C for the subsequent experimental steps of total RNA extraction. This procedure helps to ensure the integrity, stability and reliability of the samples, and to obtain high-quality results in subsequent molecular biology experiments.

### 2.3. Library Construction and On-Board Sequencing

After extracting the total RNA of the sample and digesting the DNA with DNase, the eukaryotic mRNA was enriched by magnetic beads with Oligo (dT) (in case of prokaryotes, the mRNA was enriched by removing the rRNA with the help of the kit); the mRNA was broken into short fragments by adding interrupting reagent, and then the first-strand cDNA was synthesized by using a random primer with six bases as a template, and the two-strand cDNA was synthesized by using two-strand synthesis system and purified by using the kit. Two-stranded cDNA was synthesized with six-base random primers, then two-stranded cDNA was synthesized by two-stranded synthesis reaction system, and double-stranded cDNA was purified by using reagent kits; the purified double-stranded cDNA was then subjected to end repair, addition of A-tail, and connection of sequencing junctions, and then fragment size was selected, and finally PCR amplification was carried out; the constructed libraries were qualified with Agilent 2100 Bioanalyzer (Santa Clara, CA, USA), and then the libraries were amplified by using Illumina (Shanghai, China). After the constructed library was quality checked by Agilent 2100 Bioanalyzer, it was sequenced using Illumina HiSeq X-ten sequencer (Shanghai, China) [20].

### 2.4. Transcriptome Gene Differential Expression and Enrichment Analysis

The data obtained from high-throughput sequencing is referred to as raw reads or raw data, which are subsequently subjected to quality control (QC) to determine the suitability of the sequencing data for subsequent analysis. After QC, clean reads are filtered and compared to the reference sequence using tophat/bowtie2. After the alignment, the distribution of reads on the reference sequence and the coverage of the reads were counted to determine whether the alignment results passed the second quality control (QC of alignment) [21,22]. If passed, a series of subsequent analyses such as gene expression, gene structure optimization, variable splicing, new transcript discovery and coding ability prediction, SNP detection, etc., were performed, and from the gene expression results, the genes differentially expressed among the samples were screened out, and based on the differentially expressed genes, clustering analysis, GO functional significance enrichment analysis and pathway significance enrichment analysis were performed [23,24,25].

When using RNA-seq data to comparatively analyze whether the same Unigene is differentially expressed in two samples, two criteria can be selected: one is FoldChange, which is the multiplicity of change in the expression level of the same Unigene in the two samples; the second is *p*-value or FDR (padjust), and the method of calculating the FDR value starts with the *p*-value of each Unigene, and then the FDR error control method was used to correct the *p*-value by multiple hypothesis testing [26,27]. The default screening condition for differences was *p* ≤ 0.05.

## 3. Results and Discussion

### 3.1. Physiological Changes in Response to by Sodium Acetate

**Figure 1:**
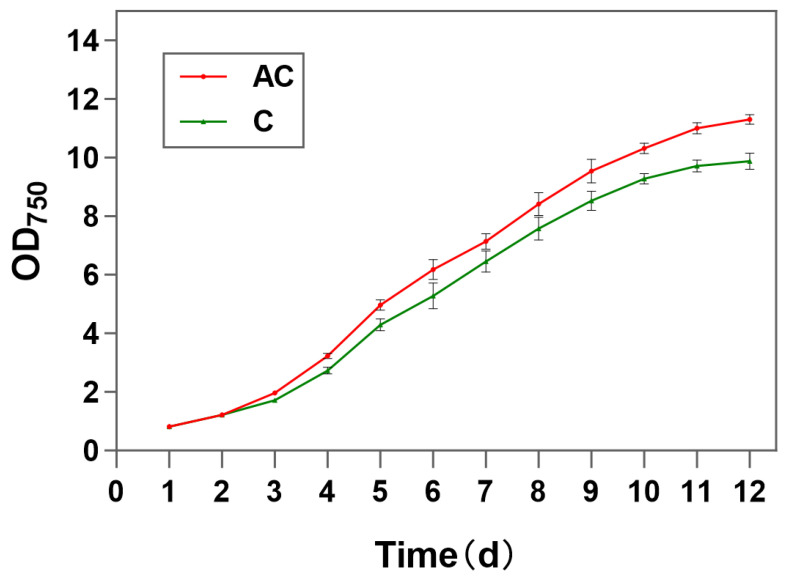
The growth curves of *N. Oceanica* in group AC and group A. “AC” represents the experimental group with sodium acetate added, and “C” represents the control group without sodium acetate added.

To assess changes in growth, physiological responses of *N. oceanica* were monitored under two distinct culture conditions: f/2 medium (C: control) and f/2 medium supplemented with 1 g/L NaAC (AC: experimental group). In this study, the growth of algae was evaluated by measuring the optical density (OD_750_) of the culture medium with a spectrophotometer. After 12 days of cultivation, significant differences in growth were observed between the control and experimental groups. The growth rate of *N. oceanica* in group AC was significantly higher than that in group A (Figure 1). It can be confirmed that sodium acetate does indeed increase the growth rate of *N. oceanica*.

### 3.2. Transcriptome Data of N. oceanica from Illumina Sequencing

The data generated from high-throughput sequencing are referred to as raw reads or raw data. To determine the suitability of these data for further analysis, quality control (QC) is essential. In this study, the assembled transcripts were used as the reference genome, and the quality-controlled sequencing reads were aligned to the reference sequences [28]. RSeQC was then employed to quantify the alignment results. This method allows for precise mapping of sequencing reads to the reference sequences, referred to as ‘mapped reads.’ Six samples were analyzed in this study, with mapped read percentages of 92.54%, 92%, 96%, 93.08%, 92.88%, 93.91%, and 92.89%, all exceeding 92% (Table 1). These results indicate that the vast majority of sequencing reads from the samples were successfully obtained and mapped, making them suitable for subsequent analysis.

Using Trimmomatic software (Version 0.39) [29] to filter the raw sequencing data is a widely used preprocessing technique that effectively removes adapter sequences and low-quality reads, resulting in clean data. These clean data are then utilized for subsequent assembly and analysis, improving alignment and assembly outcomes. The quality assessment of sequencing data reveals Q30 values for each sample in both the control and experimental groups as follows: 92.67%, 92.71%, 92.93%, 92.7%, 92.68%, and 92.68%. All values are above 92%, indicating that approximately 99.9% of the bases were accurately sequenced, with an error rate below 0.5%. The GC content of the samples ranges from 54.74% to 55.03% (Table 2). The GC content range is relatively narrow. However, it remains within the typical range observed in similar studies and does not introduce significant bias. These results demonstrate excellent data quality, ensuring a reliable basis for further analysis. The filtered and cleaned data will provide dependable support for subsequent analyses.

### 3.3. Gene Expression Distribution Analysis

**Figure 2:**
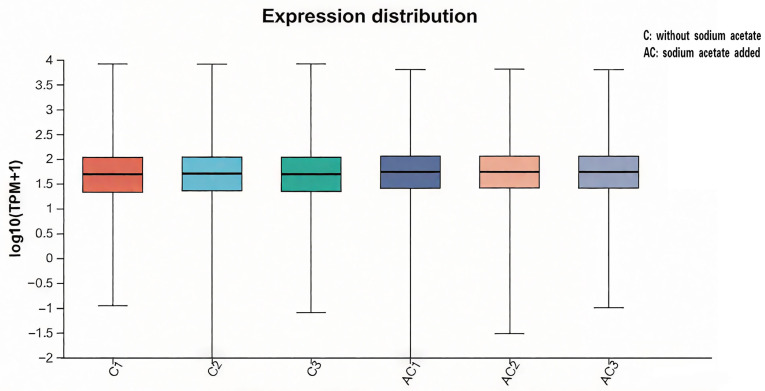
**Boxplot of gene expression distribution.** Each boxplot represented the minimum and maximum values, lower quartile, median and upper quartile of the expressed gene in the log10 TPM non-abnormal range in different samples. “AC” represents the experimental group with sodium acetate added, and “C” represents the control group without sodium acetate added.

The boxplot, a widely used tool for data visualization, allows for the comparison of gene expression distributions across different conditions. The statistical results of this study (Figure 2) indicate that there are no significant differences in the median, as well as the height and shape of the box, between the experimental group (sodium acetate added, referred to as AC) and the control group (no sodium acetate added, referred to as C). This suggests that there is no significant variation in the gene expression distribution between the two groups, implying that gene expression levels remain relatively stable under both conditions and are not significantly influenced by either treatment.

### 3.4. Identification of DEGs Under Sodium Acetate Conditions in N. oceanica

**Figure 3:**
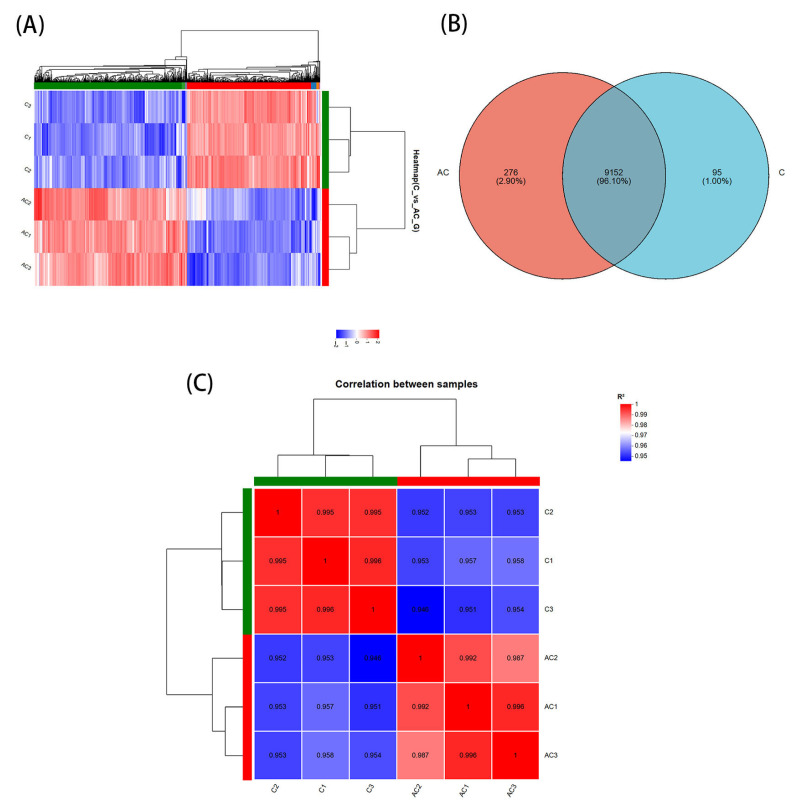
**Identification of Differentially Expressed Genes (DEGs) under sodium acetate conditions in *N. oceanica*.** (**A**) Heatmap analysis of DEGs across different treatments. Red indicates upregulation, while blue indicates downregulation. “AC” refers to the experimental group, and “C” refers to the control group. (**B**) Venn diagram comparing the experimental group (AC) with the control group (C). The numbers in the circles represent the total number and percentage of genes, while the overlapping regions show the number of common gene sequences between the experimental and control groups. (**C**) Correlation heatmap between samples. Red indicates high correlation, while blue indicates low correlation; “AC” is the experimental group and “C” is the control group.

To further explore the molecular mechanisms under sodium acetate treatment, the differential expression unigenes (DEGs) in the AC group (sodium acetate added) were compared to the C group (control, without sodium acetate). The analysis revealed a high correlation coefficient (R^2^ > 0.98) among the biological replicates within each experimental group of *N. oceanica*. This indicates that the data is both reliable and accurate, effectively reflecting the differences in gene expression of *N. oceanica* under the two treatment conditions [30] (Figure 3A).

The relationships between different DEG groups are depicted in a Venn diagram (Figure 3B), which shows a total of 9523 DEGs identified. Of these, 9152 are shared between the two groups, accounting for 96.10% of the total. To visually compare the transcriptomes between the different treatments, a heatmap was generated to display the transcript abundance of all DEGs. Hierarchical clustering of the DEGs from different treatments reveals a clear distinction in expression patterns between the control group (C) and the experimental group (AC) (Figure 3C). This analysis demonstrates that the gene expression of *N. oceanica* undergoes significant changes following the addition of sodium acetate.

Differential gene expression analysis was performed between the AC and C groups, identifying a total of 747 differentially expressed genes, including 399 upregulated genes and 348 downregulated genes (Filtering Criteria: q-value < 0.05 and |Fold Change| > 2) [31]. The results demonstrated distinct gene expression patterns between the experimental and control groups of *N. oceanica*, further indicating that the presence of sodium acetate led to significant changes in gene expression in *N. oceanica*.

### 3.5. Functional Enrichment of Differential Expressed Gene by GO and KEGG

**Figure 4:**
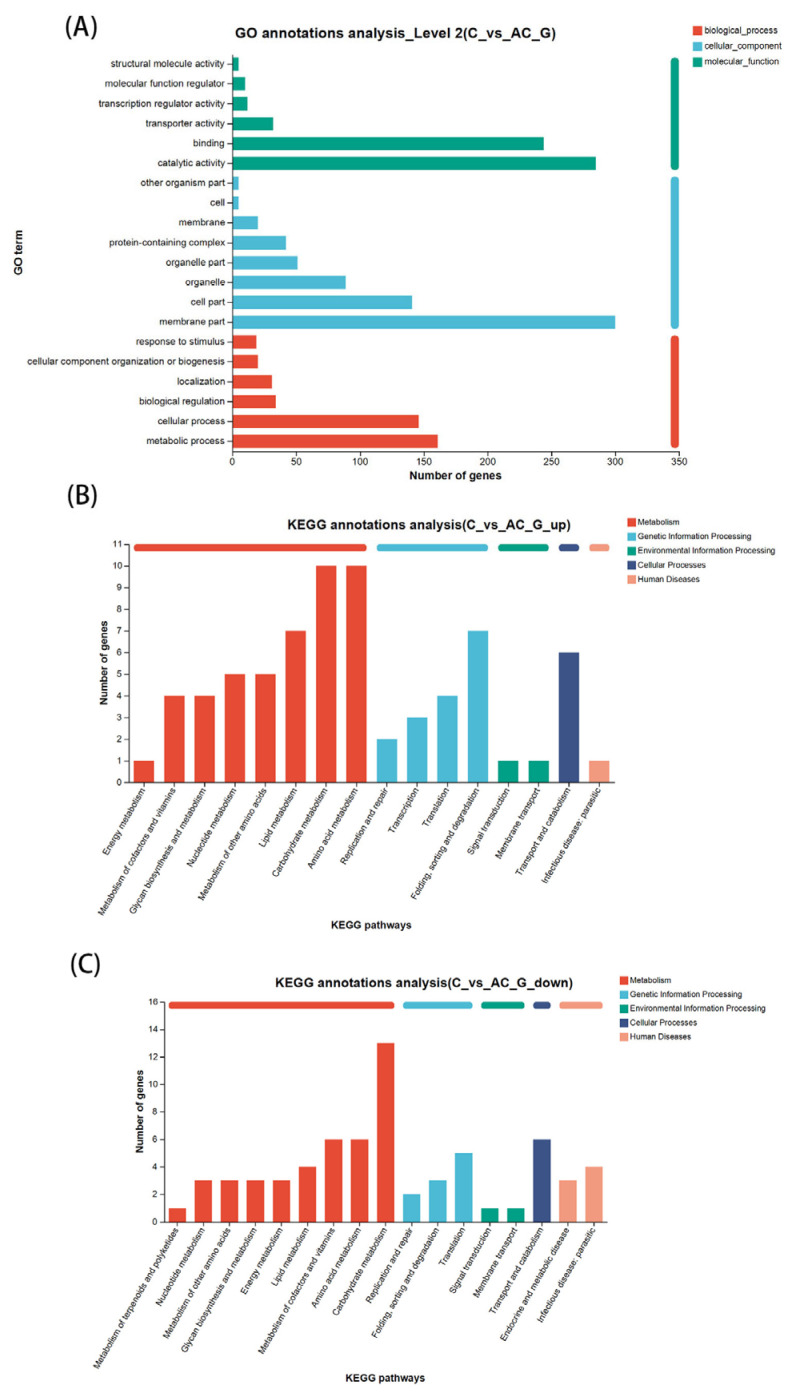
**Functional enrichments and classifications of Differentially Expressed Genes (DEGs) in *N. oceanica*.** (**A**) Gene ontology (GO) functional annotation. The figure displays the top 20 functions with the highest number of associated genes across three major categories. The horizontal axis under each column represents the number of genes annotated with each GO function. (**B**) Upregulation distribution across KEGG pathways. KEGG pathway annotations were obtained for 71 protein-coding genes, with each column representing the number of genes in the respective secondary classification pathway. The columns are color-coded in five categories, from left to right: environmental information processing, human disease, genetic information processing, cellular processes, and metabolism. (**C**) Downregulation distribution across KEGG pathways. KEGG pathway annotations were obtained for 64 protein-coding genes, with each column representing the number of genes in the secondary classification pathway. The columns are color-coded in the same five categories: environmental information processing, human disease, genetic information processing, cellular processes, and metabolism.

Gene ontology (GO) classification annotation was performed on the identified genes. As shown in Figure 4A, the DEGs were categorized into three main categories: cellular component, biological process, and molecular function [32]. The GO enrichment analysis revealed that the DEGs are predominantly associated with membrane-related functions, metabolic processes, and catalytic activities, all of which are essential for the growth, metabolism, and physiological regulation of organisms. This indicates that the addition of sodium acetate significantly influences the growth, metabolism, and physiological regulatory processes of *N. oceanica*. These findings lay a solid foundation for subsequent detailed analysis of the DEGs.

KO annotation was performed on the differential genes, and based on the relationship between KO and pathways, the KEGG metabolic pathways were classified into five main categories (Figure 4B). Enrichment analysis was conducted for each pathway using KEGG to identify those significantly enriched among the DEGs, with a *p*-value threshold of less than 0.05. Pathways that met this criterion were considered significantly enriched in DEGs. Compared to the control group, upregulated DEGs were significantly enriched in 16 pathways (Figure 4B), while downregulated DEGs were significantly enriched in 17 pathways [33] (Figure 4C) (21). Among the upregulated DEGs, the pathways with the highest number of differentially expressed genes and significant enrichment were ‘Carbohydrate metabolism’ and ‘Amino acid metabolism’ (Figure 4B). Downregulated DEGs were notably enriched in pathways such as ‘Carbohydrate metabolism’, ‘Transport and catabolism’, and ‘Translation’ (Figure 4C), with ‘Carbohydrate metabolism’ being the pathway with the highest number of differentially expressed genes and significant enrichment.

The results from KEGG annotation and functional enrichment analysis of differential genes indicate that the addition of sodium acetate significantly impacts the metabolic pathways and biological processes of *N. oceanica*. The upregulated DEGs are notably enriched in pathways related to metabolism, particularly in carbohydrate metabolism and amino acid metabolism. This suggests that sodium acetate influences *N. oceanica*’s ability to process carbohydrates and amino acids, which are essential for cellular metabolism and growth. Interestingly, carbohydrate metabolism is also the most significantly enriched pathway for downregulated DEGs, further supporting the idea that sodium acetate significantly affects carbohydrate processing. This apparent paradox reflects a profound restructuring of the carbon metabolic network rather than its blanket enhancement or repression. This reprogramming likely serves to prioritize the allocation of acetate-derived carbon toward biosynthetic and growth-related pathways, while minimizing carbon loss through oxidative processes such as the TCA cycle, thereby maximizing biomass yield. Overall, the KEGG annotation and functional enrichment analysis provide strong evidence that sodium acetate alters the metabolic and physiological processes of *N. oceanica*, with a particular emphasis on carbohydrate metabolism. These findings offer a foundation for further detailed analysis of specific differential genes.

### 3.6. The Impact of Adding Sodium Acetate on the Lipid Metabolism of N. oceanica

**Figure 5:**
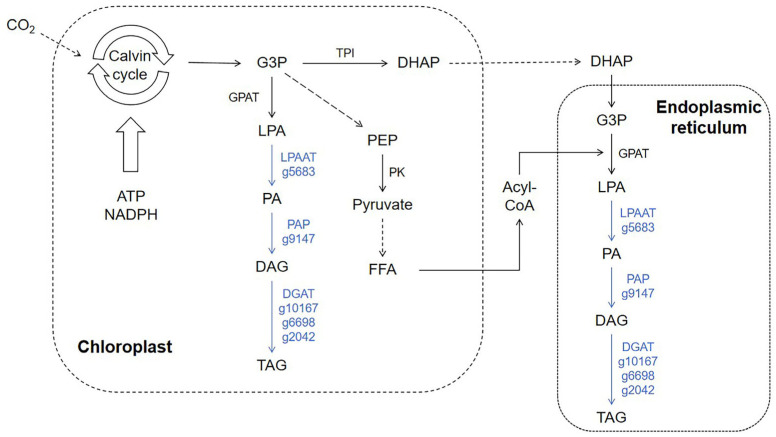
Differential expression analysis of lipid metabolism-related genes in *N. oceanica* under sodium acetate addition conditions. Differentially expressed genes were mapped onto a lipid metabolism pathway chart, with arrows indicating changes in gene transcription. Arrows of different colors represent varying transcriptional changes: “blue arrows” indicate downregulation of gene expression under sodium acetate addition conditions.

To investigate the impact of sodium acetate addition on the molecular mechanisms underlying oil production and carbon fixation in *N. oceanica*, we focused on lipid metabolic processes and annotated the relevant genes in the transcriptome. Genes encoding key enzymes in lipid metabolism, such as lysophosphatidic acid acyltransferase (LPAAT; g5683), phosphatidate phosphatase (PAP; g9147), and diacylglycerol acyltransferase (DGAT; g2042, g6698, g10167), were all significantly downregulated (Figure 5). Notably, lysophosphatidic acid acyltransferase (g5683), a critical rate-limiting enzyme in the triglyceride assembly process in plants [34], showed a 5.7-fold decrease in expression levels. Similarly, the expression of phosphatidate phosphatase (g9147) dropped significantly by 13.8-fold (Figure 5 and Table 3). Additionally, genes involved, or potentially involved, in encoding diacylglycerol acyltransferase (g2042, g6698, g10167) also exhibited varying degrees of downregulation in expression.

Glycerol 3-phosphate undergoes esterification at the sn-1 position by glycerol-3-phosphate acyltransferase (GPAT) to form lysophosphatidic acid (LPA), which is further catalyzed by lysophosphatidic acid acyltransferase (LPAAT) at the sn-2 position to form phosphatidic acid (PA) (Figure 5). PA serves as a common precursor for the synthesis of phospholipids, glycolipids, storage lipids, and extracellular lipids, playing a crucial role in lipid metabolic processes [35]. Furthermore, PA acts as a signaling molecule and is widely involved in regulating growth, development, and stress responses [36]. PA can be converted to diacylglycerol (DAG) through dephosphorylation, catalyzed by phosphatidic acid phosphatase (PAP) [37]. PAP, which catalyzes the formation of DAG from PA (Figure 5), is a key regulator of PA and DAG levels, controlling lipid metabolism, signal transduction, and playing an essential role in growth, development, and stress responses [38]. However, the expression of genes related to PAP was significantly downregulated following the addition of sodium acetate, which negatively impacts oil production (Figure 5 and Table 3). Subsequently, DAG can be catalyzed by monogalactosyldiacylglycerol (MGDG) synthase and digalactosyldiacylglycerol (DGDG) synthase to form the corresponding glycolipids, MGDG and DGDG. Additionally, DAG can be further acylated at the sn-3 position by diacylglycerol acyltransferase to form the storage lipid triacylglycerol (TAG) [39]. TAG plays an important role as an energy reserve and transport substance in plants and animals and can also serve as an indicator of an organism’s oil production capacity [40]. Lysophosphatidic acid acyltransferase, phosphatidate phosphatase, and diacylglycerol acyltransferase are all crucial enzymes in this lipid metabolic process [37,41]. Previous studies have shown that the addition of sodium acetate can increase the intracellular fatty acid content in *C. reinhardtii* and *H. pluvialis*. Based on this, we predicted that adding sodium acetate to marine *N. oceanica* would increase TAG content and enhance the expression of related genes. However, the transcriptome data did not support this prediction. In fact, the expression of several key transferase genes involved in lipid metabolism showed a significant downregulation in comparison to the control group without sodium acetate addition.

### 3.7. The Impact of Adding Sodium Acetate on Carbon Metabolism in N. oceanica

**Figure 6:**
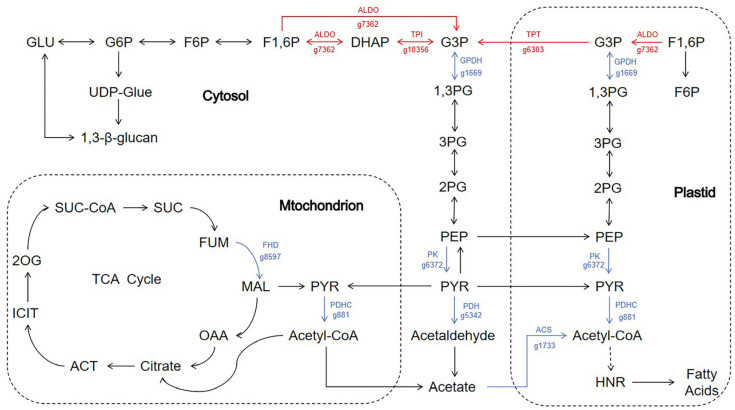
Differential expression analysis of carbon metabolism genes in *N. oceanica* under sodium acetate supplementation conditions. Differentially expressed genes were annotated on a simplified carbon metabolic pathway diagram, with arrows representing transcriptional changes. Arrows in different colors indicate varying levels of gene expression changes, with “red arrows” signifying upregulation and “blue arrows” signifying downregulation under sodium acetate supplementation.

Glycolysis is a primary pathway through which microalgae obtain energy [42]. Glyceraldehyde-3-phosphate (G3P), a key product of the Calvin cycle, is converted into pyruvate (a precursor for fatty acid biosynthesis) via the glycolysis pathway [43]. In the plastid, only the lower portion of the glycolysis pathway (from G3P to pyruvate) occurs, with the plastid fructose-1,6-bisphosphate aldolase (ALDO) catalyzing the conversion of fructose 1,6-bisphosphate (F1,6P) to G3P [44] (Figure 6). In the more upstream portion of the glycolytic pathway in the cytosol (from glucose to G3P) in *N. oceanica*, transcript levels of several G3P-related enzymes and transporters, including triosephosphate/phosphate translocator (TPT; g6303), fructose-1,6-bisphosphate aldolase (ALDO; g7362), and triphosphate isomerase (TPI; g10356), were all found to be upregulated to varying degrees (Figure 6 and Table 4). Specifically, TPT expression increased by 5.2-fold, ALDO by 7.9-fold, and TPI by 4.1-fold (Table 4). These results suggest that more G3P is being produced, which is beneficial for lipid production and aligns with previous findings to some extent [45]. Triosephosphate isomerase, also known as glyceraldehyde-3-phosphate dehydrogenase, is a well-known glycolytic enzyme that interconverts the 3-carbon sugar phosphates dihydroxyacetone (DHAP) and G3P. TPI is essential for the net production of ATP from DHAP catabolism and anaerobic glucose metabolism [46].

In addition to the genes previously mentioned, we identified several other differentially expressed genes when studying the effects of sodium acetate on the glycolytic pathway, including those encoding pyruvate kinase (PK; g6372), glycerol-3-phosphate dehydrogenase (GPDH; g1669), and pyruvate dehydrogenase (PDH; g5342) (Figure 6). Interestingly, unlike the genes encoding ALDO, TPT, and TPI, which are part of the upstream glycolytic pathway and show an upregulation trend, the genes encoding PK, GPDH, and PDH, which are involved in the downstream part of the glycolytic pathway, exhibit a downregulation trend (Figure 6 and Table 4). Pyruvate kinase, which catalyzes the conversion of phosphoenolpyruvate and ADP to ATP and pyruvate, is one of the primary rate-limiting enzymes in glycolysis [47]. Under sodium acetate treatment, pyruvate kinase expression in *N. oceanica* decreased by 10-fold (Table 4). Both glycerol-3-phosphate dehydrogenase and pyruvate dehydrogenase are crucial enzymes in glycolysis, an essential part of the energy metabolism pathway [48]. The expression of glycerol-3-phosphate dehydrogenase and pyruvate dehydrogenase in *N. oceanica* was reduced by 4.3-fold and 154.2-fold, respectively, upon sodium acetate addition (Table 4). Glycerol-3-phosphate dehydrogenase catalyzes the oxidation and phosphorylation of glycerol-3-phosphate to form 1,3-bisphosphoglycerate, which is central to glucose metabolism and plays an important role in sugar metabolism. Meanwhile, pyruvate dehydrogenase is a key enzyme linking glycolysis to the tricarboxylic acid cycle. The downregulation of genes encoding glycerol-3-phosphate dehydrogenase and pyruvate dehydrogenase impedes the oxidation of glycerol-3-phosphate to dihydroxyacetone phosphate, disrupting both glucose and lipid metabolism [49].

In addition, we observed a downward trend in the expression of genes related to fumarate hydratase (FHD) in the citric acid cycle. Fumarate hydratase is a key enzyme in the cellular TCA cycle, catalyzing the interconversion of fumarate and malate. However, the transcriptional expression of FHD (g8597) decreased by 16.5-fold after the addition of sodium acetate compared to the control group (Figure 6 and Table 4). Pyruvate can be converted into acetyl-CoA for fatty acid biosynthesis by the pyruvate dehydrogenase complex (PDHC). Experimental results showed that the transcriptional expression of the PDHC-related gene (g881) in *N. oceanica* also exhibited a downward trend, decreasing by 5.2-fold (Figure 6 and Table 4). Acetyl-CoA is a critical intermediate metabolite in energy metabolism and plays a central role in the energy metabolism of algae, suggesting that the impact of sodium acetate on *N. oceanica* is multi-dimensional. Moreover, we found that the transcriptional expression of acetyl-CoA synthetase (ACS; g1733) significantly decreased, with a reduction of up to 31.4-fold (Table 4). This further highlights the broad impact of sodium acetate on key metabolic processes in *N. oceanica*.

In summary, *N. oceanica* responded to the addition of sodium acetate through multilevel metabolic reprogramming. In the glycolytic pathway, the expression of upstream genes encoding ALDO, TPT, and TPI was significantly upregulated (4.1–7.9-fold), while the expression of downstream genes encoding PK, GPDH, and PDH was significantly decreased (7.4–154.2-fold). Notably, genes encoding fumarate hydratase (FHD) in the tricarboxylic acid cycle were also downregulated, leading to a dual regulation of “anterior glycolysis activation-TCA cycle inhibition.” This cross-pathway synergistic regulation pattern suggests that *N. oceanica* may enhance the upstream metabolic flux of glycolysis to produce pentose phosphate intermediates, while inhibiting the allocation of pyruvate to the downstream carbon flow of the TCA cycle. This results in carbon bridging and redirection at the transcriptional level, which serves to ensure the immediate production of NADPH/ATP while reducing acetyl-CoA levels, thereby maintaining energy metabolic homeostasis by minimizing carbon loss in the acetyl-CoA oxidation pathway.

### 3.8. The Impact of Adding Sodium Acetate on Fatty Acid Synthesis and Lipase in N. oceanica

In order to investigate the effects of sodium acetate addition on fatty acid synthesis and lipase-related impacts, we conducted a detailed analysis of genes associated with fatty acid synthesis and esterase in *N. oceanica*. The results of the analyses were unlike previous studies which found that sodium acetate maximised the total fatty acid content of *C. reinhardtii* [10]. Most of the genes associated with fatty acid synthesis and esterases, including those related to fatty acid synthase, lysophospholipase, esterase and lipase, showed varying degrees of down-regulation under conditions of sodium acetate addition (Table S1). This is not consistent with previous analyses on the impact of sodium acetate addition on lipid metabolism in *N. oceanica*. The discrepancy with Yang’s results may be attributed to the fact that they added sodium acetate under phosphorus and nitrogen deficiency conditions, which could have altered the carbon fixation strategy of *C. reinhardtii* [10]. Additionally, it is noteworthy that while most genes related to fatty acid synthesis and esterase (including g927, g481, g9804, g5370, g9755, g272, g1028, g10055, g1019, g1615, g2137, g471, g1989, g8602, g272, g5463) showed downregulation at the transcriptional level, there were still genes related to esterase, lipase, and thioesterase family proteins (g7143) that exhibited a certain degree of upregulation (Table S1). These results suggest that the metabolic pathways associated with fatty acid synthesis and esterase activity in *N. oceanica* are somewhat altered in the presence of sodium acetate addition. There have been similar studies before; sodium acetate has been found to not only affect the growth of algal cells but also regulate lipid and carotenoid-related metabolic processes. Specifically, lipid and carotenoid contents of *Chlorella sorokiniana* increased 2.4-fold and 1.2-fold, respectively, under the addition of 3.0 g/L sodium acetate [50]. However, contrary to our expectations, sodium acetate does not lead to an increase in the transcriptional levels of genes associated with fatty acid synthesis and lipid production in *N. oceanica*. In fact, the majority of genes related to fatty acid synthesis, esterase, and lipid metabolism in *N. oceanica* tend to be downregulated under the condition of adding sodium acetate. This discrepancy may be attributed to profound phylogenetic divergence among different microalgal lineages. *Chlamydomonas* (Chlorophyta) and *Haematococcus* (Chlorophyta) are evolutionarily distinct from *N. oceanica* (Eustigmatophyceae), which likely results in fundamental differences in their core carbon metabolic networks and regulatory logic in response to organic carbon sources. Additionally, many studies reporting sodium acetate-induced lipid accumulation were conducted under nutrient stress conditions (nitrogen or phosphorus deprivation), which itself strongly triggers lipid accumulation as a carbon storage mechanism. In contrast, our study was performed under nutrient-replete conditions. Thus, the physiological scenario of “nutrient sufficiency + organic carbon” likely activates a distinct regulatory program in *N. oceanica*, favoring the preferential utilization of acetate for rapid proliferation rather than energy storage.

### 3.9. The Impact of Adding Sodium Acetate on Carbon Fixation in N. oceanica

To investigate the impact of sodium acetate supplementation on carbon fixation in *N. oceanica*, we examined the transcripts of genes associated with the Calvin Cycle, CCM (Carbon Concentration Mechanism), and other processes related to carbon and fixation. Among these, only the gene encoding fructose-1,6-bisphosphate aldolase (ALDO; g7362) showed a slight upregulation, while most other Calvin Cycle-related genes exhibited varying degrees of downregulation, including the gene encoding ribulose-phosphate 3-epimerase (RPE; g5017), which was downregulated by 19.8-fold (Table S2). Ribulose-phosphate 3-epimerase plays a crucial role in the Calvin Cycle, as it catalyzes the interconversion between xylulose 5-phosphate (Xu5P) and ribulose 5-phosphate (Ru5P), an important step in the cycle due to its involvement in the isomerization of sugar phosphates, which is essential for maintaining the balance of metabolic intermediates required for the cycle’s function [51]. Under the condition of adding sodium acetate, the expression of the gene encoding ribulose-phosphate 3-epimerase (g5017) decreased (Table S2), which to some extent indicates a weakening of the Calvin Cycle following the addition of sodium acetate. This would lead to a reduction in the accumulation of carbon compounds in *N. oceanica*, resulting in a decrease in its carbon sequestration capacity. Previous research has demonstrated that a reduction in the expression of ribulose-phosphate 3-epimerase can decrease carbon fixation in Arabidopsis chloroplasts, leading to a feedback inhibition of photosynthetic electron transport and ATP synthase activity due to photosynthetic control, thereby reducing the photosynthetic capacity of *Arabidopsis* [52]. Consistent with these findings, the experimental group *N. oceanica* also found a reduction in the expression of ATPase-related genes following the addition of sodium acetate. This suggests that the photosynthetic capacity of *N. oceanica* may be compromised to some extent following the addition of sodium acetate. The decrease in expression of RPE (g5017) in *N. oceanica* further supports this hypothesis, indicating a potential reduction in carbon fixation and photosynthetic efficiency. Fructose 1,6 bisphosphate aldolase is a crucial enzyme involved in the Calvin Cycle of photosynthesis. It plays a significant role in controlling the operation of photosynthesis by catalyzing the reversible cleavage of fructose 1,6-bisphosphate into dihydroxyacetone phosphate (DHAP) and glyceraldehyde 3-phosphate (G3P) [53]. FBA has been shown to respond differently under various stress conditions and is capable of binding to the cytoskeleton, participating in microtubule aggregation, endocytosis, and vesicle transport [54]. Additionally, it is involved in the infection process of pathogens. It is one of the few key enzymes that have shown an upward trend in gene expression following the addition of sodium acetate, which is noteworthy. Overexpression of FBA increases the production of unsaturated fatty acids in yeast, thereby improving the efficiency of biodiesel production [55]. This has implications for our future efforts to genetically engineer *N. oceanica* to increase the yield of unsaturated fatty acids.

Regarding the CCM, carbonic anhydrase (CA), which is responsible for the conversion between carbon dioxide and bicarbonate, is annotated as a key component of the CCM [56,57]. Contrary to expectations, the gene encoding carbonic anhydrase (g2018) was downregulated 8.69-fold upon sodium acetate addition (Table S2). This suggests that sodium acetate regulates the CCM at the RNA level, implying that sodium acetate weakens the microalgae’s carbon concentrating mechanism, which also involves metabolic processes where organic carbon participates in promoting biological growth. Unfortunately, we have not annotated candidate genes responsible for bicarbonate transport, which is another key component of the CCM [58].

Under the condition of adding sodium acetate, the expression of the gene encoding ribulose-phosphate 3-epimerase (g5017) decreased (Table S2), which indicates a significant suppression of carbon fixation capacity in the Calvin cycle. In the presence of a readily available organic carbon source (sodium acetate), maintaining a highly active Calvin cycle would consume substantial amounts of light energy and reducing power (ATP and NADPH). The pronounced downregulation of this pathway serves as an energy-saving strategy, allowing the cell to redirect finite energy resources away from carbon fixation and toward more urgent biosynthetic processes—such as upstream glycolysis—to support the observed rapid growth. This finding is consistent with the overarching pattern observed throughout our study: “reducing internal carbon production while enhancing external carbon utilization.” It corroborates previously noted downregulation of photosynthesis-related genes and key components of the carbon concentrating mechanism (carbonic anhydrase, CA). Together, these results paint a coherent picture: the cell is actively reducing investment in autotrophic metabolism in favor of prioritizing the use of exogenous acetate as its primary carbon source for biosynthesis.

In addition to this, we found a trend of suppressed expression of genes related to acetyl coenzyme a, such as acyl-CoA synthetases (g1733) and long chain acyl-CoA synthetase (g1617). Their expression was down-regulated 31.4-fold and 6.5-fold (Table S2), respectively. It is well known that acetate can be converted to acetyl-CoA by acetyl-CoA synthetase and then enter the glyoxylate cycle or tricarboxylic acid cycle, where ATP and NAD(P)H produced are used for algal growth and biosynthesis [59]. While the gene encoding acetyl CoA synthase showed a down-regulation trend, which affects both the glyoxylate cycle and the tricarboxylic acid cycle, we have previously found that the expression of some related genes (g8597, g881) in the tricarboxylic acid cycle also showed a down-regulation trend after the addition of sodium acetate (Table S2), and the two trends showed the same trend. This suggests that sodium acetate does affect *N. oceanica* in the tricarboxylic acid cycle.

### 3.10. Photosynthesis Affected in Response to Sodium Acetate Addition

To investigate the impact of sodium acetate supplementation on the photosynthesis of *N. oceanica*, we conducted a detailed screening of transcripts related to Photosystem I and II, as well as genes involved in chlorophyll biosynthesis. Unfortunately, we only identified significant differential expression in two photosynthesis-related genes: light harvesting complex protein 3 (g9514) and phytol kinase (g1121). After adding sodium acetate, the expression levels of both genes encoding light harvesting complex protein 3 (g9514) and phytol kinase (g1121) in *N. oceanica* showed a significant downward trend, with decreases of 34.2-fold and 43.6-fold, respectively. This suggests that the photosynthetic activity of *N. oceanica* may be inhibited. This is consistent with our previous discussion, as well as the findings of Meloni, who discovered that the downregulation of the gene encoding Ribulose-phosphate 3-epimerase can have a certain inhibitory effect on photosynthesis [51].

In addition, acetate was found to inhibit photosynthesis in the green microalga *Chlorella sorokiniana*. Under conditions where acetate is added, the activity of Photosystem I and Photosystem II is adversely affected to varying degrees, resulting in a trend of decreased photosynthetic activity [60]. In *H. pluvialis*, sodium acetate has been shown to promote the tricarboxylic acid cycle, the glyoxylate cycle (TCA), and lipid synthesis, while inhibiting activities associated with photosynthesis, thereby accelerating algal growth and astaxanthin accumulation [61]. This further corroborates our hypothesis that sodium acetate indeed has an inhibitory effect on the photosynthesis of *N. oceanica*. However, it was not found in *N. oceanica* that the tricarboxylic acid cycle could get boosted after the addition of sodium acetate, suggesting that it may adopt a different adaptive mechanism than *H. pluvialis*.

In conclusion, although sodium acetate did inhibit the photosynthesis of *N. oceanica*, this is not entirely in conflict with the significant increase in the growth rate of *N. oceanica* observed. As a readily available source of both organic carbon and energy, sodium acetate can be directly utilized by the cells. We propose that the pronounced downregulation of photosynthesis—an energy-intensive process requiring constant investment of light energy and the construction and maintenance of extensive photosynthetic machinery—represents an efficient resource-saving strategy. By reallocating finite resources from de novo carbon production toward the utilization of immediately available carbon for biosynthesis, the cell maximizes its growth rate. When organic carbon is abundant, *N. oceanica* actively reduces investment in its “internal factory” and preferentially exploits the more efficient “external supply,” reflecting an adaptive decision that enhances its competitiveness and fitness.

## 4. Conclusions

Using mRNA-Seq technology, we systematically investigated the response of *N. oceanica* to sodium acetate addition, offering new insights into the regulatory mechanisms involved. Critically, despite the observed widespread downregulation of essential processes, our physiological data demonstrate that this transcriptional response occurred concurrently with a significant enhancement in growth rate. At the transcriptional level, we observed significant reprogramming characterized by a strategic downregulation of processes that become “uneconomical” or redundant when organic carbon is available (photosynthesis, carbon fixation, and storage lipid synthesis). This extensive downregulation is not a random occurrence but a hallmark of active cellular regulation, constituting a form of “strategic attenuation” wherein *N. oceanica* actively abandons a self-sustaining autotrophic economy to prioritize the use of readily available environmental resources.

Conversely, pathways more directly supporting biosynthesis and growth (such as upper glycolysis) were upregulated. This distinct “trade-off” pattern aligns with the principle of resource optimization. These findings enhance our understanding of metabolic reprogramming under sodium acetate treatment and the regulation of the associated metabolites, demonstrating that the organism employs selective sacrifice of non-essential functions to reallocate precious cellular resources toward processes that maximize immediate growth rate and competitive fitness. This study provides a valuable reference for future in-depth studies on the effects of sodium acetate on *N. oceanica*.

## Figures and Tables

**Table 1:** Compare the results to the statistical.

Sample	Total Reads	Total Mapped
C1	19,791,288	18,315,060 (92.54%)
C2	15,363,420	14,281,880 (92.96%)
C3	16,002,066	14,894,103 (93.08%)
AC1	18,369,538	17,061,148 (92.88%)
AC2	18,476,094	17,239,512 (93.91%)
AC3	17,748,090	17,239,512 (93.91%)

**Table 2:** Statistical table of sequencing data.

Sample	Raw Reads	Raw Bases	Clean Reads	Clean Bases	Error Rates (%)	Q20 (%)	Q30 (%)	GC Content (%)
C1	20,078,104	3,031,793,704	19,791,288	2,956,576,263	0.0267	97.24	92.67	54.74
C2	15,592,272	2,354,433,072	15,363,420	2,299,530,630	0.0267	97.27	92.71	54.89
C3	16,247,216	2,453,329,616	16,002,066	2,386,477,217	0.0264	97.35	92.93	54.78
AC1	18,598,860	2,808,427,860	18,369,538	2,745,983,768	0.0267	97.26	92.7	55.02
AC2	18,774,698	2834,979,398	18,476,094	2,756,361,229	0.0267	97.25	92.68	55.03
AC3	18,024,086	2,721,636,986	17,748,090	2,652,452,542	0.0267	97.25	92.68	54.98

**Table 3:** Differential gene expression participated in lipid metabolism in *N. oceanica*.

Gene ID	Gene Name	Abbreviation	Fold Change (AC vs. C; Fold)
g5683	Acyltransferase	LPAAT	5.7 ↓
g9147	PA-phosphatase-like phosphoesterase	PAP	13.8 ↓
g10167	Mono-or diacylglycerol acyltransferase type 2	DGAT	5.1 ↓
g6698	Diacylglycerol acyltransferase, putative		5.9 ↓
g2042	Type 2 diacylglycerol acyltransferase		6.9 ↓

“↓” represented down-regulation.

**Table 4:** Differential gene expression participated in carbon metabolism and lipid metabolism in *N. oceanica*.

Gene ID	Gene Name	Abbreviation	Fold Change (AC vs. C; Fold)
g7362	Fructose-1,6-bisphosphate aldolase	ALDO	7.9 ↑
g10356	Triosephosphate isomerase	TPI	4.1 ↑
g6303	Triosephosphate/phosphate translocator	TPT	5.2 ↑
g8597	Fumarate hydratase class II	FHD	16.5 ↓
g881	Dihydrolipoamide acetyltransferase, pyruvate dehydrogenase complex component E2	PDHC	5.2 ↓
g1733	Acetyl CoA synthetase	ACS	31.4 ↓
g5342	Pyruvate dehydrogenase	PDH	154.22 ↓
g1669	Glycerol-3-phosphate dehydrogenase	GPDH	4.3 ↓
g6372	Pyruvate kinase	PK	10.0 ↓
g10046	Pyruvate dehydrogenase kinase	PDK	7.4 ↓

“↑” and “↓” represented up- and down-regulation, respectively.

## Data Availability

Relevant data is included in the article and Supplementary Materials.

## Supplementary Materials

The following supporting information can be downloaded at: https://www.mdpi.com/article/10.3390/life15091398/s1, Supplemental Dataset S1: Differential expression genes under two different conditions in *N. oceanica*. Table S1: Differential gene expression related to fatty acid synthesis and lipase in *N. oceanica.* Table S2: Differential gene expression related to and carbon fixation in *N. oceanica.* Figure S1: Pre-experimental growth curve. Supplementary document: *N. oceanica* medium and specific operation methods.

## Abbreviations

The following abbreviations are used in this manuscript:

DEGs	Differential expression unigenes
G3P	Glyceraldehyde-3-phosphate
LPA	Lysophosphatidic acid
PA	Phosphatidic acid
GPAT	Glycerol-3-phosphate acyltransferase
DAG	Diacylglycerol
TAG	Triacylglycerol
LPAAT	Lysophosphatidic acid acyltransferase
PAP	Phosphatidate phosphatase
DGAT	Diacylglycerol acyltransferase
F6P	Fructose 6-phosphate
F1,6P	Fructose 1,6-bisphosphate
DHAP	Phosphates dihydroxyacetone
PEP	Phosphoenolpyruvate
PYR	Pyruvate
FUM	Fumarate
MAL	Malate
ALDO	Fructose-1,6-bisphosphate aldolase
TPI	Triphosphate-isomerase
TPT	Triosephosphate/phosphate translocator
GPDH	Glycerol-3-phosphate dehydrogenase
PK	Pyruvate kinase
PDH	Pyruvate dehydrogenase
FHD	Fumarate hydratase
PDHC	Pyruvate dehydrogenase complex
ACS	Acetyl-CoA synthetase

## References

[1] Yun H.S., Kim Y.S., Yoon H.S. (2021). Effect of Different Cultivation Modes (Photoautotrophic, Mixotrophic, and Heterotrophic) on the Growth of *Chlorella* sp. and Biocompositions. Front. Bioeng. Biotechnol..

[2] Li Y., Tian W., Fu Z., Ye W., Zhang X., Zhang Z., Sun D. (2022). Mechanisms of Sodium-Acetate-Induced DHA Accumulation in a DHA-Producing Microalga, *Crypthecodinium* sp. SUN. Mar. Drugs.

[3] Liu J., Huang J., Fan K.W., Jiang Y., Zhong Y., Sun Z., Chen F. (2010). Production potential of *Chlorella zofingienesis* as a feedstock for biodiesel. Bioresour. Technol..

[4] Tambat V.S., Patel A.K., Singhania R.R., Vadrale A.P., Tiwari A., Chen C.W., Dong C.D. (2023). Sustainable mixotrophic microalgae refinery of astaxanthin and lipid from Chlorella zofingiensis. Bioresour. Technol..

[5] Lu X., Zhao W., Wang J., He Y., Yang S., Sun H. (2024). A comprehensive review on the heterotrophic production of bioactive compounds by microalgae. World J. Microbiol. Biotechnol..

[6] Chen J., Chen Y., He W., Liang H., Hong T., Li T., Du H. (2024). Transcriptome analysis reveals the molecular mechanism of differences in growth between photoautotrophy and heterotrophy in *Chlamydomonas reinhardtii*. Front. Plant Sci..

[7] Hu Q., Song M., Huang D., Hu Z., Wu Y., Wang C. (2021). *Haematococcus pluvialis* Accumulated Lipid and Astaxanthin in a Moderate and Sustainable Way by the Self-Protection Mechanism of Salicylic Acid Under Sodium Acetate Stress. Front. Plant Sci..

[8] Ramanan R., Kim B.-H., Cho D.-H., Ko S.-R., Oh H.-M., Kim H.-S. (2013). Lipid droplet synthesis is limited by acetate availability in starchless mutant of *Chlamydomonas reinhardtii*. FEBS Lett..

[9] Yee W. (2015). Feasibility of various carbon sources and plant materials in enhancing the growth and biomass productivity of the freshwater microalgae *Monoraphidium griffithii* NS16. Bioresour. Technol..

[10] Yang L., Chen J., Qin S., Zeng M., Jiang Y., Hu L., Xiao P., Hao W., Hu Z., Lei A. (2018). Growth and lipid accumulation by different nutrients in the microalga *Chlamydomonas reinhardtii*. Biotechnol. Biofuels.

[11] Byeon H., An Y., Kim T., Rayamajhi V., Lee J., Shin H., Jung S. (2023). Effects of Four Organic Carbon Sources on the Growth and Astaxanthin Accumulation of *Haematococcus lacustris*. Life.

[12] Senousy H.H., El-Sheekh M.M., Khairy H.M., El-Sayed H.S., Mahmoud G.A., Hamed A.A. (2023). Biodiesel Production from the Marine Alga *Nannochloropsis oceanica* Grown on Yeast Wastewater and the Effect on Its Biochemical Composition and Gene Expression. Plants.

[13] Canini D., Martini F., Cazzaniga S., Miotti T., Pacenza B., D’Adamo S., Ballottari M. (2024). Genetic engineering of *Nannochloropsis oceanica* to produce canthaxanthin and ketocarotenoids. Microb. Cell Fact..

[14] Brennan B., Regan F. (2020). In-situ lipid and fatty acid extraction methods to recover viable products from *Nannochloropsis* sp. Sci. Total Environ..

[15] Liu M., Yu L., Zheng J., Shao S., Pan Y., Hu H., Shen L., Wang W., Zhou W., Liu J. (2024). Turning the industrially relevant marine alga *Nannochloropsis* red: One move for multifaceted benefits. New Phytol..

[16] Chen X., Khatiwada J.R., Chio C., Shrestha S., Kognou A.L.M., Fan L., Qin W. (2024). Low-cost cultivation of *Nannochloropsis oceanica* in newly designed photobioreactors and its productivity trends in semi-continuous cultivation under inland outdoor conditions. Bioresour. Technol..

[17] Ma X.-N., Chen T.-P., Yang B., Liu J., Chen F. (2016). Lipid Production from *Nannochloropsis*. Mar. Drugs.

[18] Lin W., Li P., Liao Z., Luo J. (2017). Detoxification of ammonium to *Nannochloropsis oculata* and enhancement of lipid production by mixotrophic growth with acetate. Bioresour. Technol..

[19] Lintner M., Balzano S., Keul N., Heinz P., Manecki M., Klimek A., Wanek W., Cyran N., Gruber D., Schmidt K. (2025). Biosorption of heavy metals by microalgae: Hazardous side effects for marine organisms. Chemosphere.

[20] Wei L., You W., Gong Y., El Hajjami M., Liang W., Xu J., Poetsch A. (2020). Transcriptomic and proteomic choreography in response to light quality variation reveals key adaption mechanisms in marine *Nannochloropsis oceanica*. Sci. Total Environ..

[21] Zhao R., Yin S.Y., Jiang C.H., Xue J.N., Liu C., Cai X.H., Xing Y.P., Kang T.G. (2022). Comparison of chloroplast genomes of medicinal plants in Aristolochiaceae. Zhongguo Zhong Yao Za Zhi.

[22] O’Neill E.A., Fehrenbach G., Murphy E., Alencar S.A., Pogue R., Rowan N.J. (2022). Use of next generation sequencing and bioinformatics for profiling freshwater eukaryotic microalgae in a novel peatland integrated multi-trophic aquaculture (IMTA) system: Case study from the Republic of Ireland. Sci. Total Environ..

[23] Kanehisa M., Goto S. (2000). KEGG: Kyoto encyclopedia of genes and genomes. Nucleic Acids Res..

[24] Soneson C., Love M.I., Robinson M.D. (2015). Differential analyses for RNA-seq: Transcript-level estimates improve gene-level inferences. F1000Research.

[25] Wang J., Chen J., Zhang D., Cui X., Zhou J., Li J., Wei Y., Bu D. (2023). Integrated Omics Approach to Discover Differences in the Metabolism of a New Tibetan *Desmodesmus* sp. in Two Types of Sewage Treatments. Metabolites.

[26] Kong S., Kubatko L.S. (2021). Comparative Performance of Popular Methods for Hybrid Detection using Genomic Data. Syst. Biol..

[27] Robertson D.S., Wason J.M.S., Ramdas A. (2023). Online multiple hypothesis testing. Stat. Sci..

[28] Albanese D., Donati C. (2021). Genome Recovery, Functional Profiling, and Taxonomic Classification from Metagenomes. Methods Mol. Biol..

[29] Sewe S.O., Silva G., Sicat P., Seal S.E., Visendi P. (2022). Trimming and Validation of Illumina Short Reads Using Trimmomatic, Trinity Assembly, and Assessment of RNA-Seq Data. Methods Mol. Biol..

[30] Yue L.I., Yunlong W., Longling O. (2022). Comparative analyses on the transcriptome among free-living *zooxanthellae* under different phosphate concentrations. J. Fish. Sci. China.

[31] Zhang X., Zhang Y., Chen Z., Gu P., Li X., Wang G. (2023). Exploring cell aggregation as a defense strategy against perchlorate stress in *Chlamydomonas reinhardtii* through multi-omics analysis. Sci. Total Environ..

[32] Mu X., Chen Y. (2021). The physiological response of photosynthesis to nitrogen deficiency. Plant Physiol. Biochem..

[33] Xu W., Zhang J., Yang C., Ai F., Yin Y., Guo H. (2024). Differential impacts of organic and inorganic phosphorus on the growth and phosphorus utilization of *Microcystis aeruginosa*. Sci. Total Environ..

[34] Wayne L.L., Gachotte D.J., Graupner P.R., Adelfinskaya Y., McCaskill D.G., Metz J.G., Zirkle R., Walsh T.A. (2021). Plant and algal lysophosphatidic acid acyltransferases increase docosahexaenoic acid accumulation at the sn-2 position of triacylglycerol in transgenic *Arabidopsis* seed oil. PLoS ONE.

[35] Duan J.L., Han Y., Liu X.Y., Liu M.Y., Sun Y.C., Ma J.Y., Sun X.D., Wang Y., Tan M.M., Gong B. (2025). Membranal phosphatidylglycerol enhances oxygen diffusion and release from cyanobacteria. Water Res..

[36] Liu Y., Yin Y., Hao H., Wang R., He Z., Tian R., Liu G. (2021). Identification of phosphatidic acid interacting proteins in Ganoderma lingzhi. Sheng Wu Gong Cheng Xue Bao.

[37] Ukey R., Carmon T., Hardman D., Hill N., Fakas S. (2020). The *Yarrowia lipolytica* PAH1 homologue contributes but is not required for triacylglycerol biosynthesis during growth on glucose. Yeast.

[38] Orive-Milla N., Delmulle T., de Mey M., Faijes M., Planas A. (2020). Metabolic engineering for glycoglycerolipids production in E. coli: Tuning phosphatidic acid and UDP-glucose pathways. Metab. Eng..

[39] Li Y., Xu J., Li G., Wan S., Batistič O., Sun M., Zhang Y., Scott R., Qi B. (2019). Protein S-acyl transferase 15 is involved in seed triacylglycerol catabolism during early seedling growth in *Arabidopsis*. J. Exp. Bot..

[40] Imbs A.B., Dembitsky V.M. (2023). Coral Lipids. Mar. Drugs.

[41] Zhou Y., Huang X., Hao Y., Cai G., Shi X., Li R., Wang J. (2022). Cloning and functional characterization of a lysophosphatidic acid acyltransferase gene from Perilla frutescens. Sheng Wu Gong Cheng Xue Bao.

[42] Zhu L., Feng S., Li Y., Sun X., Sui Q., Chen B., Qu K., Xia B. (2024). Physiological and transcriptomic analysis reveals the toxic and protective mechanisms of marine microalga *Chlorella* pyrenoidosa in response to TiO(2) nanoparticles and UV-B radiation. Sci. Total Environ..

[43] Gurrieri L., Fermani S., Zaffagnini M., Sparla F., Trost P. (2021). Calvin-Benson cycle regulation is getting complex. Trends Plant Sci..

[44] Sobanski T., Suraweera A., Burgess J.T., Richard I., Cheong C.M., Dave K., Rose M., Adams M.N., O’Byrne K.J., Richard D.J. (2023). The fructose-bisphosphate, Aldolase A (ALDOA), facilitates DNA-PKcs and ATM kinase activity to regulate DNA double-strand break repair. Sci. Rep..

[45] Xue L.-L., Chen H.-H., Jiang J.-G. (2017). Implications of glycerol metabolism for lipid production. Prog. Lipid Res..

[46] Myers T.D., Palladino M.J. (2023). Newly discovered roles of triosephosphate isomerase including functions within the nucleus. Mol. Med..

[47] Schormann N., Hayden K.L., Lee P., Banerjee S., Chattopadhyay D. (2019). An overview of structure, function, and regulation of pyruvate kinases. Protein Sci. A Publ. Protein Soc..

[48] Jeon S., Baek H., Kim S., Kim Y., Kim J., Kim J.W. (2025). Microalgae-Derived Microparticles Improve Immunomodulation via Combined Glycolysis and MAPK Activation. Langmuir.

[49] Driver T., Trivedi D.K., McIntosh O.A., Dean A.P., Goodacre R., Pittman J.K. (2017). Two Glycerol-3-Phosphate Dehydrogenases from *Chlamydomonas* Have Distinct Roles in Lipid Metabolism. Plant Physiol..

[50] Liu Q., Hu X., Yang Y., Dong J., Gao Z., Qian P., Deng X. (2021). Growth and metabolites of *Chlorella sorokiniana* regulated by sodium acetate. Microbiol. China.

[51] Meloni M., Fanti S., Tedesco D., Gurrieri L., Trost P., Fermani S., Lemaire S.D., Zaffagnini M., Henri J. (2024). Characterization of chloroplast ribulose-5-phosphate-3-epimerase from the microalga *Chlamydomonas reinhardtii*. Plant Physiol..

[52] Li Y.H., Peng L.W., Wang X.Q., Zhang L. (2022). Reduction in chloroplastic ribulose-5-phosphate-3-epimerase decreases photosynthetic capacity in *Arabidopsis*. Front. Plant Sci..

[53] Liu X., Gao Y., Tang S., Ben L., Zhang X., Dong G., Zhou J., Lin L., Yang Z., Zhou Y. (2025). A Dual-localized Fructose Bisphosphate Aldolase is Essential for Chloroplast Development and Carbon Metabolism in Rice. Rice.

[54] Zhang C.-S., Hawley S.A., Zong Y., Li M., Wang Z., Gray A., Ma T., Cui J., Feng J.-W., Zhu M. (2017). Fructose-1,6-bisphosphate and aldolase mediate glucose sensing by AMPK. Nature.

[55] He S.-C., Zhang Z.-Y., Han Y.-Q., Miao L., Zhang C.-Y., Yu A.-Q. (2024). Research Progress in the Production of Polyunsaturated Fatty Acids by Yarrowia lipolytica Cell Factories. Biotechnol. Bull..

[56] Jensen E.L., Maberly S.C., Gontero B. (2020). Insights on the Functions and Ecophysiological Relevance of the Diverse Carbonic Anhydrases in Microalgae. Int. J. Mol. Sci..

[57] Malerba M.E., Marshall D.J., Palacios M.M., Raven J.A., Beardall J. (2021). Cell size influences inorganic carbon acquisition in artificially selected phytoplankton. New Phytol..

[58] Zhu H., Ye Z., Xu Z., Wei L. (2024). Transcriptomic Analysis Reveals the Effect of Urea on Metabolism of *Nannochloropsis oceanica*. Life.

[59] Lauersen K.J., Willamme R., Coosemans N., Joris M., Kruse O., Remacle C. (2016). Peroxisomal microbodies are at the crossroads of acetate assimilation in the green microalga *Chlamydomonas reinhardtii*. Algal Res..

[60] Liu K., Li J., Qiao H., Lin A., Wang G. (2012). Immobilization of *Chlorella sorokiniana* GXNN 01 in alginate for removal of N and P from synthetic wastewater. Bioresour. Technol..

[61] Yu X., Ye X., Hu C., Xu N., Sun X. (2022). Sodium acetate can promote the growth and astaxanthin accumulation in the unicellular green alga *Haematococcus pluvialis* as revealed by a proteomics approach. J. Oceanol. Limnol..

