# Supplementary material for: Transcriptomic Survey of How Acetate Addition Affected the Growth in Nannochloropsis oceanica (Suda & Miyashita) R. E. Lee
Source: Life (Basel). 2025 Sep 3;15(9):1398. doi: 10.3390/life15091398 (PMC12471308; doi:10.3390/life15091398)
Supplement: Supplementary file 1 [file life-15-01398-s001.zip › Supplementary documentú║N.oceanica medium and specific operation methods.pdf]

N. oceanica f/2 medium and specific operation methods:

The f/2 medium is formulated as follows： 35 g/L sea salt (Real Ocean, USA), 1 g/L $NaNO_3$, 3.65 mg/L $FeCl_3$*$6H_2O$, 67 mg/L NaH_2PO_4*H_2O, 4.37 mg/L Na_2EDTA*$2H_2O$, trace metal mix (0.36 mg/L $MnCl_2$*$4H_2O$, 0.0126 mg/L $NaMoO_4$*$2H_2O$, 0.0196 mg/L, $CuSO_4$*$5H_2O$, 0.044 mg/L $ZnSO_4$*$7H_2O$, , and 0.01mg/L $CoCl_2$), and vitamin mix (2.5 μg/L biotin, 2.5 μg/L VB12, and 0.5 μg/L thiamine HCl) . Make f/2 medium before seawater with diaphragm vacuum pump filtration before adding other formulations. After the components were fully dissolved, put them into an autoclave and sterilize at 121℃ for 20 min. On the ultra-clean bench, 50 mL of algal liquid of *N. oceanica* was taken, and the supernatant was discarded after centrifugation at 5000×g for 5 min to obtain the algal sludge, 5 mL of which was resuspended in the medium, and then inoculated into a conical flask containing 200 mL of f/2 medium, and then placed on a shaking table (150 r/min) at 24-26 ℃ for 24 h of incubation, with continuous illumination, and the intensity of the illumination was 70-100 μmol/(m^2-s). Repeat the above steps for 3 consecutive rounds of activation culture to obtain the pure species of *N. oceanica*. The subsequent experiments were carried out.
