# Supplementary material for: Transcriptomic Survey of How Acetate Addition Affected the Growth in Nannochloropsis oceanica (Suda & Miyashita) R. E. Lee
Source: Life (Basel). 2025 Sep 3;15(9):1398. doi: 10.3390/life15091398 (PMC12471308; doi:10.3390/life15091398)
Supplement: Supplementary file 1 [file life-15-01398-s001.zip › Table S1. Differential gene expression related to fatty acid synthesis and lipase in N. oceanica.pdf]

Table S1. Differential gene expression related to fatty acid synthesis and lipase in *N. oceanica.*

Gene ID	Gene name	Fold change (U VsCt; fold)
g927	Type I fatty acid synthase,putative	7.3↓
g481	Stearoyl-ACP desaturase	13.0↓
g9804	Similar to lysophospholipase	5.1↓
g5370	Hormone-sensitive lipase,putative	6.2↓
g9755	PREDICTED: lysophospholipase-like 1	6.0↓
g272	Ab hydrolase superfamily protein;lysophospholipase	9.1↓
g1028	5-nucleotidase/2,3-cyclic phosphodiesterase family esterase	4.0↓
g7143	Esterase/lipase/thioesterase family protein	6.7↑
g10055	Esterase	4.0↓
g1019	Phosphatidylglycerophosphate synthase 1	11.3↓
g1615	Fatty acid elongase	29.6↓
g2137	Diacylglycerol kinase	4.3↓
g471	Phosphoglycerate dehydrogenase/Phosphoserine aminotransferase	21.9↓

g1989	Alpha/beta fold family hydrolase/acetyltransferase	12.1↓
g8602	Hydrolase,alpha/beta fold family protein	10.1↓
g272	Ab hydrolase superfamily protein;lysophospholipase	9.1↓
g5463	Alpha/beta hydrolase	8.1↓

"↑" and "↓" represented up- and down-regulation respectively
