# Supplementary material for: Transcriptomic Survey of How Acetate Addition Affected the Growth in Nannochloropsis oceanica (Suda & Miyashita) R. E. Lee
Source: Life (Basel). 2025 Sep 3;15(9):1398. doi: 10.3390/life15091398 (PMC12471308; doi:10.3390/life15091398)
Supplement: Supplementary file 1 [file life-15-01398-s001.zip › Table S2. Differential gene expression related to and carbon fixation in N. oceanica.pdf]

Table S2. Differential gene expression related to and carbon fixation in *N. oceanica.*

Gene ID	Gene name	Fold change (U VsCt; fold)
g1855	Boron transporter 1	5.04↓
g7362	Fructose-1,6-bisphosphate aldolase	7.9↑
g2018	Carbonic anhydrase	8.69↓
g1455	Phosphatidylinositol phosphatase	38.6↓
g1965	Inositol-3-phosphate synthase	6.9↓
g521	Triose or hexose phosphate/phosphate translocator	19.8↓
g9805	Acyl-CoA dehydrogenase	85.28↓
g182	Mitochondrial carrier;citrate transporter	6.2↓
g1965	Inositol-3-phosphate synthase	6.9↓
g5471	Phosphatidylglycerol/phosphatidylinositol transfer protein precursor	22.4↓
g5017	Ribulose-phosphate 3-epimerase	19.8↓
g6303	Triosephosphate/phosphate translocator	2.3↑
g1616	Carotenoid oxygenase	30.3↓

g1617	Long chain acyl-CoA synthetase	6.5↓
g8609	Aurora-like Serine/threonine protein kinase	26.9↓
g8463	Beta-glucosidase	29.9↓
g9160	Glucosamine 6-phosphate synthetase	12.2↓
g5893	Glutamine-fructose-6-phosphate transaminase	2.8↑
g2817	Endoglucanase	2.1↑
g9662	Cellulase 2;endo-1,4-beta-glucanase	4.0↓
g7102	ATP-binding cassette transporter,subfamily I,member 9,ABC component protein PpABCI9	2.4↑
g1007	ATP-binding cassette transporter	2.6↑
g171	Phospholipid-transporting ATPase 3	24.6↓
g1230	Phospholipid-transporting ATPase DNF1	14.7↓
g1733	Acetyl CoA synthetase	31.4↓

"↑" and "↓" represented up- and down-regulation respectively
