# Supplementary figures and images for: Transcriptomic Survey of How Acetate Addition Affected the Growth in Nannochloropsis oceanica (Suda & Miyashita) R. E. Lee
Source: Life (Basel). 2025 Sep 3;15(9):1398. doi: 10.3390/life15091398 (PMC12471308; doi:10.3390/life15091398)

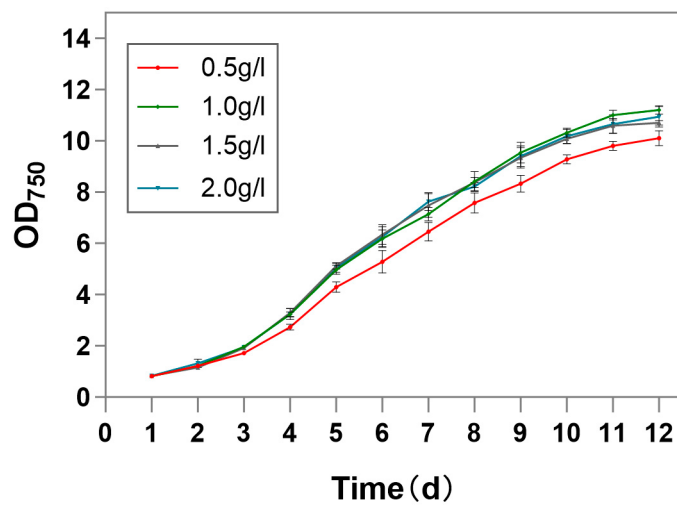

**Figure S1.** Pre-experimental growth curve.

Supplement: Supplementary file 1 [file life-15-01398-s001.zip › Figure S1. Pre-experimental growth curve..pdf]
